# Supplementary material for: Intestinal Cells-on-Chip for Permeability Studies
Source: Micromachines (Basel). 2024 Nov 30;15(12):1464. doi: 10.3390/mi15121464 (PMC11679574; doi:10.3390/mi15121464)
Supplement: Supplementary file 1 [file micromachines-15-01464-s001.zip › micromachines-3317795-supplementary.pdf]

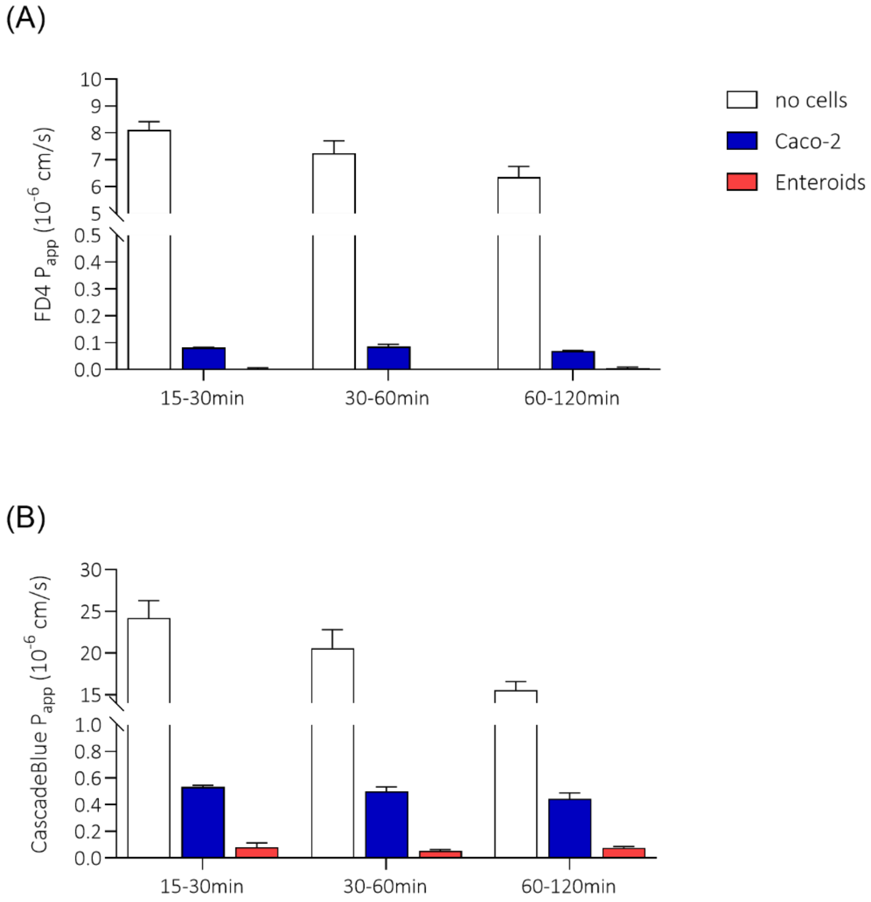

Supplementary Figure S1: Epithelial barrier integrity in static Transwell controls. Apparent permeability (P_{app}) values for (A) dextran-FITC 4kDa (FD4) and (B) Cascade Blue 0.5 kDa (CB) in intestinal cell monolayers on Transwell inserts, measured at three intervals, including no cell control inserts. n=3 per group

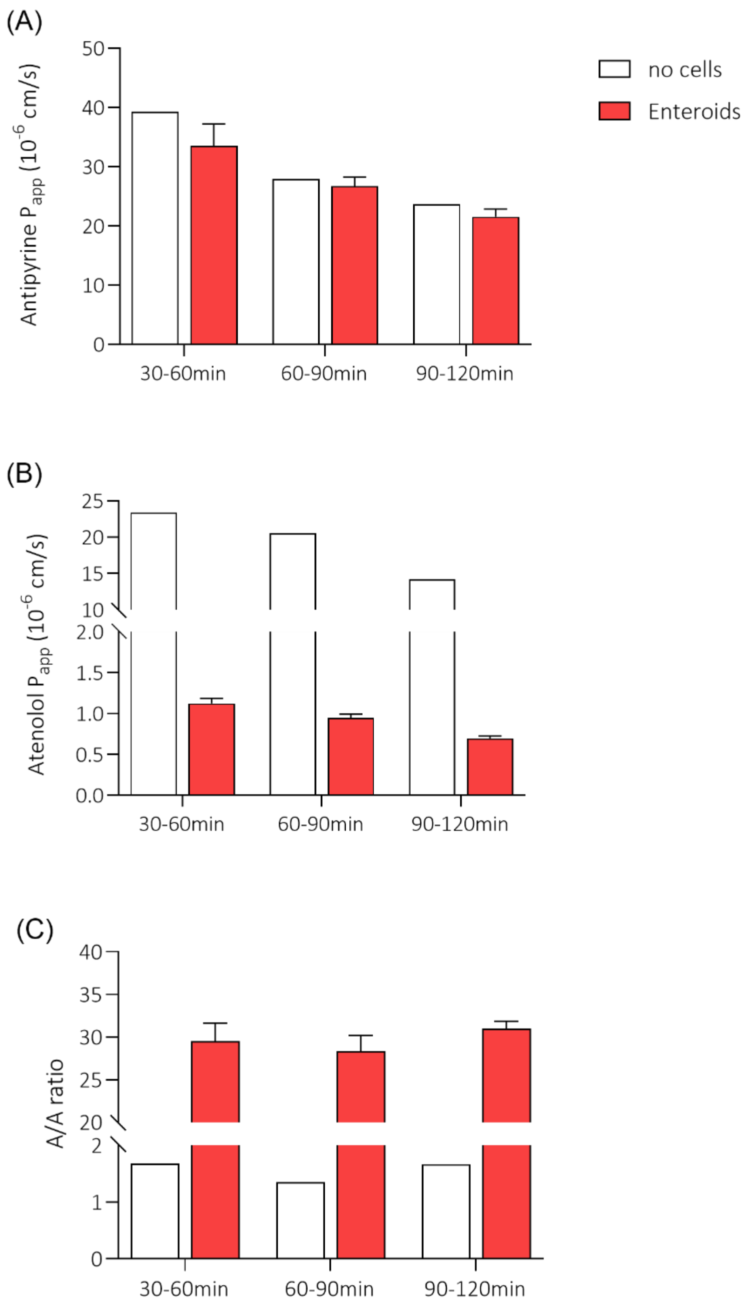

Supplementary Figure S2: Epithelial barrier permeability in static Transwell controls. Apparent permeability (P_{app}) values for (A) antipyrine and (B) atenolol in enteroid monolayers on Transwell inserts measured at three intervals, including no cell control inserts, with (C) corresponding A/A ratios. n=4 for enteroids, with a single no cell control insert.

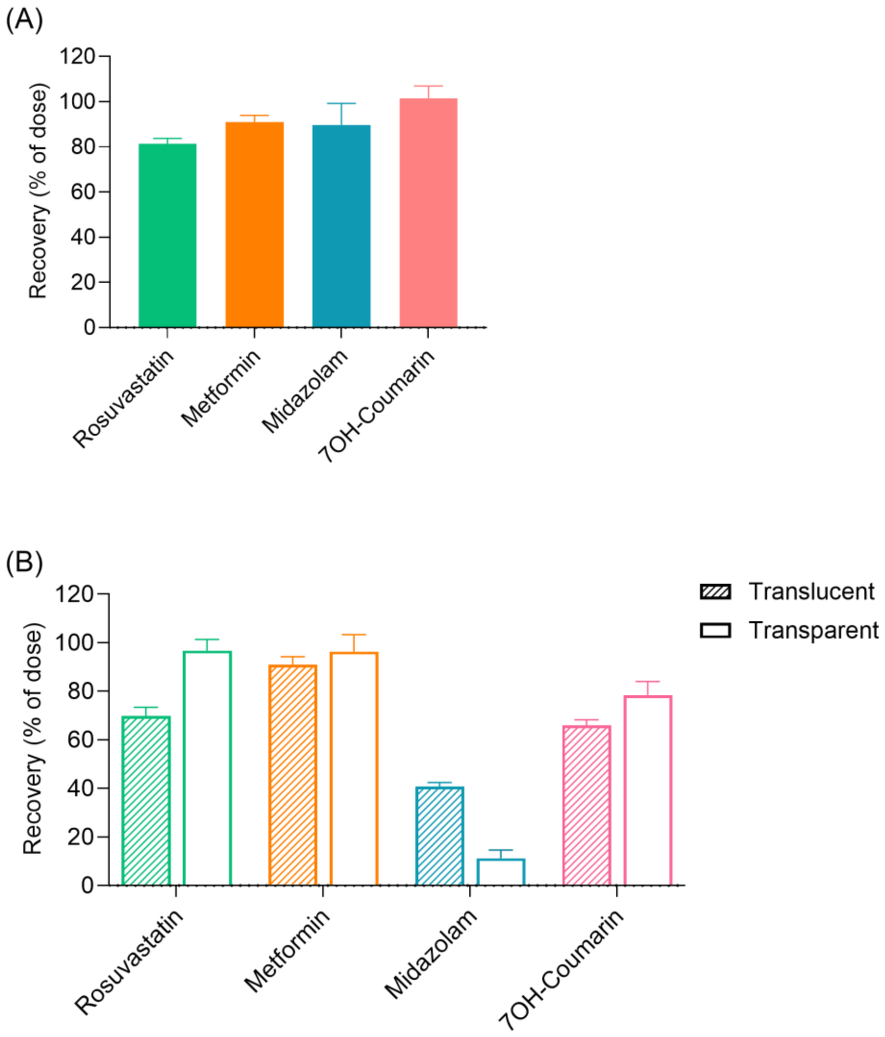

Supplementary Figure S3: Drug recovery. Recovery of 4 different compounds in (A) static Transwell experiments and (B) chip experiments with both translucent and transparent membranes, expressed as percentage of the original dose added. n=4-6 per group.
